# Adherence to life’s essential 8 is associated with delayed white matter aging

**DOI:** 10.1016/j.ebiom.2025.105723

**Published:** 2025-04-24

**Authors:** Li Feng, Zhenyao Ye, Yezhi Pan, Rozalina G. McCoy, Braxton D. Mitchell, Peter Kochunov, Paul M. Thompson, Jie Chen, Menglu Liang, Thu T. Nguyen, Edmond Shenassa, Yan Li, Travis Canida, Hongjie Ke, Hwiyoung Lee, Song Liu, L. Elliot Hong, Chixiang Chen, David K.Y. Lei, Shuo Chen, Tianzhou Ma

**Affiliations:** a Department of Nutrition and Food Science, College of Agriculture & Natural Resources, University of Maryland, College Park, MD, USA; b Department of Epidemiology and Biostatistics, School of Public Health, University of Maryland, College Park, MD, USA; c Maryland Psychiatric Research Center, Department of Psychiatry, School of Medicine, University of Maryland, Baltimore, MD, USA; d Division of Biostatistics and Bioinformatics, Department of Epidemiology and Public Health, School of Medicine, University of Maryland, Baltimore, MD, USA; e Division of Endocrinology, Diabetes, and Nutrition, Department of Medicine, School of Medicine, University of Maryland, Baltimore, MD, USA; f Division of Gerontology, Department of Medicine, School of Medicine, University of Maryland, Baltimore, MD, USA; g University of Maryland Institute for Health Computing, North Bethesda, MD, USA; h Department of Health Policy and Management, School of Public Health, University of Maryland, College Park, MD, USA; i Division of Endocrinology, Diabetes, & Nutrition, Department of Medicine, School of Medicine, University of Maryland, Baltimore, MD, USA; j Louis A. Faillace Department of Psychiatry & Behavioral Sciences, McGovern Medical School, University of Texas Health Science Center at Houston, Houston, TX, USA; k Imaging Genetics Center, Keck School of Medicine, University of Southern California, Marina del Rey, CA, USA; l Maternal & Child Health Program, School of Public Health, University of Maryland, College Park, MD, USA; m Department of Epidemiology, School of Public Health, Brown University, RI, USA; n Department of Mathematics, The College of Computer, Mathematical, and Natural Sciences, University of Maryland, College Park, MD, USA; o School of Computer Science and Technology, Qilu University of Technology (Shandong Academy of Sciences), Jinan, Shandong, China

**Keywords:** Life’s essential 8, White matter, Brain age gap, Machine learning, *APOE4*

## Abstract

**Background:**

The American Heart Association introduced Life’s Essential 8 (LE8) to promote cardiovascular health and longevity. However, its impact on brain ageing and interactions with genetic risk factors of dementia, such as *APOE4*, remains unclear. This study investigates the relationship between LE8 and white matter brain ageing and evaluates the moderating effects of the *APOE4* allele.

**Methods:**

This cross-sectional study utilized data from the UK Biobank, including genetic, neuroimaging, and health-related data from touchscreen questionnaires, physical examinations, and biological samples. Participants were non-pregnant whites with LE8 variables, diffusion tensor imaging (DTI) data, and *APOE4* genetic information available, excluding those with extreme white matter hyperintensities. Regional fractional anisotropy measures from DTI data were used to predict white matter brain age via random forest regression. The white matter brain age gap (BAG) was calculated by subtracting chronological age from predicted brain age.

**Findings:**

The analysis included 18,817 participants (9430 women and 9387 men; mean age 55.45 years [SD: 7.46]). Higher LE8 scores were associated with a lower white matter BAG, indicating delayed brain ageing. The effect was more pronounced in non-*APOE4* carriers (124 days younger per 10-point increase, 95% CI: 102–146 days; p < 0.001) compared to *APOE4* carriers (84 days younger per 10-point increase, 95% CI: 47–120 days; *p* < 0.001). Potential interaction between *APOE4* and LE8 on brain ageing was observed for some age and sex groups but with only borderline significance, further investigation in larger and more targeted studies is needed to validate the finding.

**Interpretation:**

Adherence to LE8 is associated with delayed brain ageing, with genetic factors such as *APOE4* potentially moderating this effect in specific age and sex groups. The overall benefit from a healthier lifestyle in individuals’ brain ageing across genetic, sex, and age groups underscore the importance and broad applicability of behavioural lifestyle interventions in promoting brain health.

**Funding:**

US National Institute of Health, 10.13039/100008510University of Maryland, Montgomery County of Maryland.

**Research in context**

**Evidence before this study**

The American Heart Association introduced Life’s Essential 8 (LE8) as a comprehensive set of eight metrics that reflect health behaviours to support cardiovascular health (CVH). Prior studies have linked LE8 to neuroimaging markers of brain health including total brain volume, white matter hyperintensities (WMH), total grey matter and hippocampal volume. However, the impact of LE8 on brain ageing at the microstructural level particularly in relation to white matter integrity, and its interaction with key genetic risk factors of Alzheimer’s disease and related dementia, such as *APOE4*, remains unclear.

**Added value of this study**

In this study, we examined LE8’s association with White Matter Brain Age Gap (WM BAG) and whether *APOE4* status modifies this relationship across age and sex groups using UK Biobank data. WM BAG is a machine-learning-derived biomarker from diffusion tensor imaging data and offers a more comprehensive and interpretable measure of structural brain ageing. As compared to other neuroimaging markers like brain volume and WMH, WM BAG is more sensitive to early and subtle change in WM integrity thus providing insights into early detection of neurodegenerative diseases, tracking of individual brain ageing and disease progression, and lifestyle-related neuroprotection. We assessed both the composite LE8 metrics and each individual lifestyle behaviour and health factor, as well as their potential Gene × Environment interaction with *APOE4*, to better understand the lifestyle influence on brain ageing, and how this could be modified by the one’s genetic susceptibility to dementia.

**Implications of all the available evidence**

Our findings underscore the role of lifestyle factors in preserving white matter integrity and slowing brain ageing, supporting LE8 as a promising modifiable factor for brain health. While *APOE4* carriers showed slightly attenuated effect as compared to non-*APOE4* carriers, our study demonstrated overall benefits from a healthier lifestyle for individuals across genetic, sex, and age groups. These results reinforce the broad applicability of lifestyle interventions in promoting brain health.

## Introduction

The American Heart Association introduced Life’s Essential 8 (LE8) as a comprehensive set of eight metrics that reflect health behaviours that support cardiovascular health (CVH),^1^ with the aim to help older individuals maintain CVH and live longer and healthier. These eight measures are categorized into two major areas: health behaviours (eating healthier foods, being more active, quitting tobacco, getting healthy sleep) and health factors (managing weight, controlling cholesterol, managing blood glucose, managing blood pressure). Beyond its association with CVH, LE8 is increasingly recognized for its impact on neurological health. Recent studies linked higher LE8 scores with neuroimaging markers of better brain health (total brain volume, white matter hyperintensities (WMH), total grey matter and hippocampal volume),^2^^,^^3^ and better brain health outcomes (better cognitive function,^2^^,^^4^^,^^5^ reduced risk of mild cognitive impairment (MCI), dementia and depression^6^^,^^7^). As people age, the brain undergoes many changes including reduced neurogenesis, impaired synaptic connections, increased inflammation and among others,^8^ which have been the major cause of cognitive decline and primary risk factor of dementia and other neurodegenerative diseases in the elderly. However, the broad and early-stage impact of LE8 on brain ageing at the microstructural level, particularly in relation to white matter integrity, remains unexplored.

‘Brain age’ aims to predict chronological age from structural or functional neuroimaging features using a machine learning algorithm.^9^ BAG, which measures the discrepancy between chronological age and brain age, has served as a precursor to cognitive decline and potential neurodegenerative diseases.^10^ Specifically, WM, the primary pathway for communication between different brain regions, is widely used to predict brain age as its structure and integrity significantly change with age. WM microstructural integrity has also been recognized as an early biomarker for the prodromal stage of Alzheimer’s disease (AD), dementia, and other neurodegenerative disorders.^11^^,^^12^ Here, we focus on age-related WM microstructural changes and employ a machine learning algorithm^9^^,^^13^^,^^14^ to predict WM brain age from regional fractional anisotropy (FA) measures derived from diffusion tensor imaging (DTI) data. As compared to other DTI derived measures, FA was previously shown as the primary factor contributing to cognitive ageing.^15^ In this study, we investigate how potential lifestyle-promoting factors like LE8 impact WM BAG. As compared to traditional neuroimaging markers like brain volume and WMH, WM BAG offers a potentially more comprehensive and interpretable measure of WM integrity. It may capture early and subtle changes in white matter structure, providing insights into early detection of certain neurodegenerative diseases, tracking of individual brain ageing and disease progression, and lifestyle-related neuroprotection.^16^

In addition to environmental and lifestyle factors, multiple genetic variants have been implicated in brain ageing and dementia risk.^17^^,^^18^ The *APOE4* allele, among the strongest prevalent genetic risk factor of AD, is associated with an increased risk for AD by promoting the accumulation of beta-amyloid plaques and tau protein abnormalities, causing inflammation, compromising vascular health, and reducing neuroprotection in the brain^19^. However, the impact of *APOE4* on brain ageing has been inconsistent across studies.^20^^,^^21^ Some research also showed that *APOE4* may mitigate the protective effect from the lifestyle factors,^22^ and sex may exhibit different effects.^23^

**Fig. 1:**
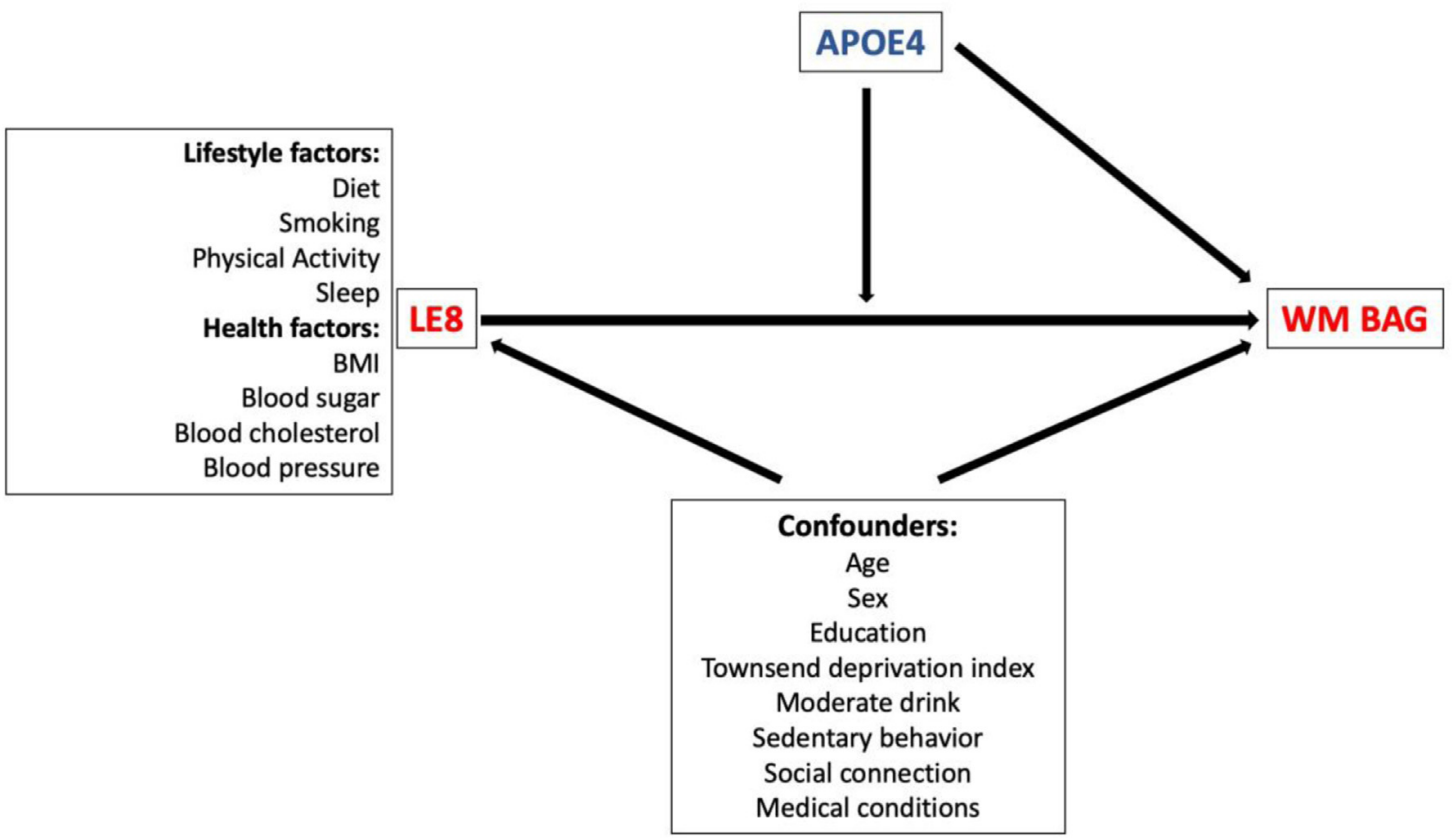
Directed acyclic graph (DAG): Illustrating the association between life’s essential 8 (LE8) and white matter brain age gap (WM BAG), controlling for confounders and modulated by the effect of *APOE4* genotype.

To fill the gap, here we conducted a study integrating genetic, neuroimaging, and health-related data from touchscreen questionnaires, physical examinations, and biological samples in UK Biobank (UKB) to investigate the effect of LE8 (overall and of each lifestyle factor separately) on WM brain ageing and examine whether these effects between LE8 and WM brain ageing are modified by *APOE4* status (a Gene × Environment interaction effect) in an age and sex-dependent manner (Fig. 1). We hypothesized that the inconsistent associations between *APOE4* and brain ageing are driven, in part, by its interaction with lifestyle factors such as LE8. These data can provide mechanistic insights into how genetics and lifestyle factors jointly influence brain ageing and cognitive impairment, ultimately informing personalized health care and prevention plans for those at risk for AD and related dementia.

## Methods

This cross-sectional study utilizes data from the UK Biobank (UKB), a population-based cohort study of more than 500,000 individuals aged 40–69 years in 22 recruitment centres across the UK, initially recruited from 2006 to 2010. The brain imaging phenotypic data collection began in 2014 for approximately 40,000 UKB participants.^24^ UK Biobank has approval from the North West Multicenter Research Ethics Committee (MREC) as a Research Tissue Bank (RTB) approval (https://www.ukbiobank.ac.uk/learn-more-about-uk-biobank/about-us/ethics).

**Fig. 2:**
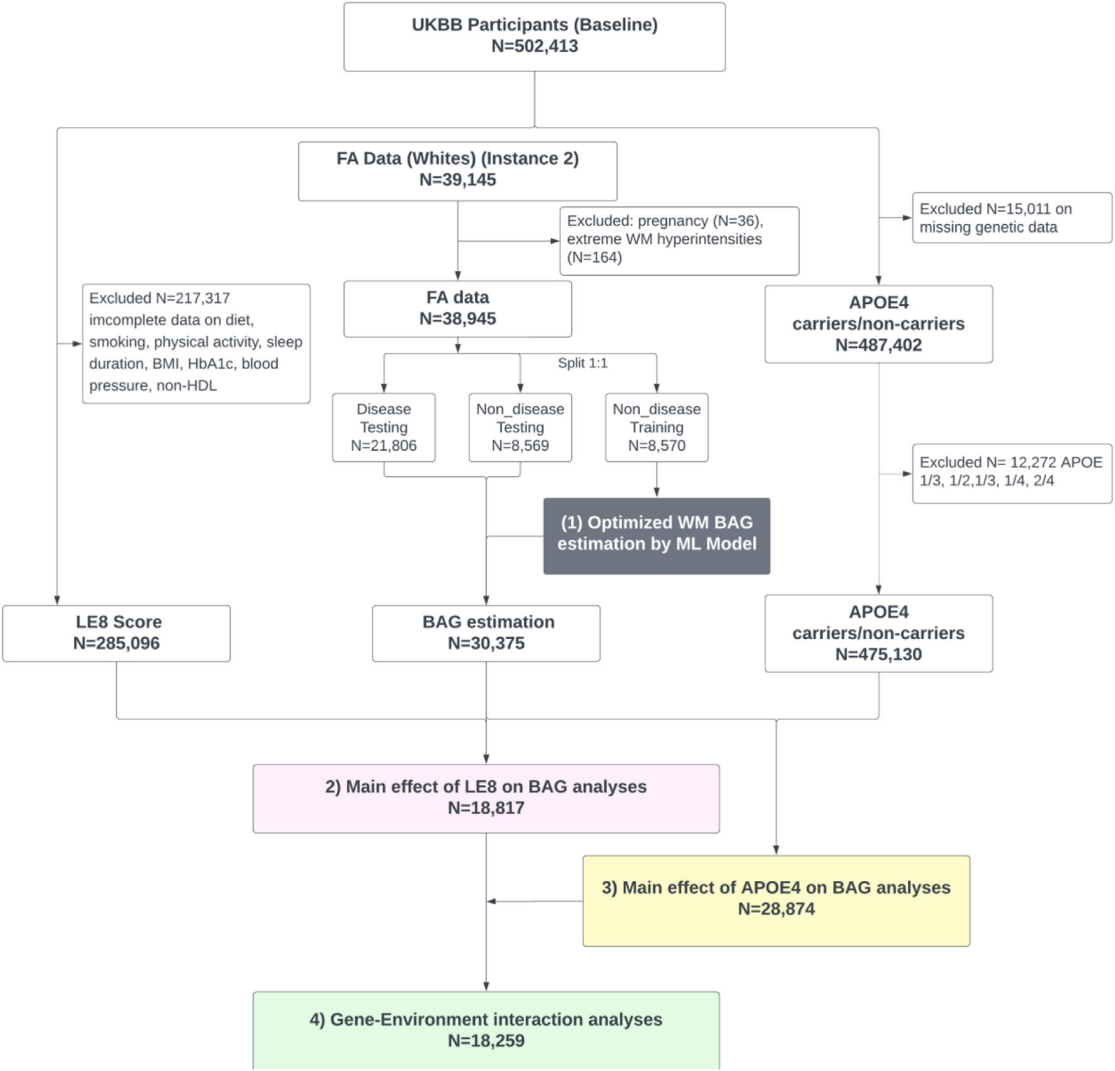
Flow chart of participants selection and data processing procedures. Flowchart of our analysis procedures and the number of subjects included at each step of the analysis. It details three key analytical processes: (1) Utilizing a machine learning model to estimate age bias-corrected white matter (WM) brain age gap (BAG) (shown in grey); (2) Analysing the linear relationship between Life’s Essential 8 (LE8) and WM BAG (highlighted in pink); (3) Analysing the main effect of *APOE4* carriers on WM BAG (highlighted in yellow); and (4) Assessing *APOE4* (gene) by LE8 (environment) interaction on WM BAG as the primary analysis (indicated in green).

We focused specifically on non-pregnant white (predominantly European) participants to avoid bias of imbalanced racial distribution in UKB cohort and reduce cross-population heterogeneity in training the brain age prediction model, consistent with our previous studies.^13^^,^^14^ We calculated the LE8 score for those with complete data on all LE8 variables available (N = 285,096). We estimated the outcome WM BAG for participants with available FA data and without extreme WMH (those with total volume of WMH above Q3 + 1.5∗IQR from T1 and T2_FLAIR images) (N = 30,375), to prevent distortions in structural brain measures.^25^ We investigated the main effect of LE8 on WM BAG among N = 18,817 participants with both data available. We further considered participants with genetic data on *APOE4* available to investigate the main effect of *APOE4* on WM BAG (N = 28,874) and the *APOE4* x LE8 interaction effect (N = 18,259). Details of the inclusion and exclusion criteria are illustrated in the flowchart (Fig. 2). Evaluation of baseline demographics indicates no systematic differences between excluded and included participants, supporting the representativeness of our final analytic sample.

### Neuroimaging data

The outcome, WM BAG, was computed based on the FA measures collected from DTI data from the UKB imaging assessment. A set of 48 standard-space tracts was used to generate tract-specific masks from skeletonized images, which were then applied to compute the mean FA for each tract per subject using the Johns Hopkins University (JHU) DTI Atlas, similar to the ENIGMA protocol (http://enigma.ini.usc.edu/protocols/dti-protocols).^26^^,^^27^ This study focuses on 39 WM FA tracts covering multiple brain regions (see the list of 39 regional WM FA measures in Supplementary Table S1). The average value for each white matter tract in the brain was computed using Tract-Based Spatial Statistics analysis applied to the DTI FA images.^28^ Water diffusion is directionally restricted in healthy white matter, and FA values, which range from 0 (completely random diffusion, indicating damaged or less structured tissue) to 1 (highly directional diffusion, reflecting intact white matter), quantify the level of anisotropy in a diffusion process and the integrity of the white matter. For robustness and completeness, we also extended our investigation beyond FA to include three additional diffusion tensor imaging (DTI) metrics: axial diffusivity (AD), mean diffusivity (MD), and radial diffusivity (RD) and evaluated their associations with the exposures.

### Assessment of Life’s essential 8

The LE8 score is aligned with guidance from the American Heart Association^1^ and comprises eight key metrics representing lifestyle behaviours (diet, physical activity, smoking, sleep) and health factors (body mass index (BMI), lipids (non-HDL cholesterol), haemoglobin A1c (HbA1c), and blood pressure (SBP and DBP). Diet quality scoring criteria are based on the Dietary Approaches to Stop Hypertension (DASH)-style diet. These eight metrics were collected and derived from health-related data from touchscreen questionnaires, physical examinations, and biological samples in UKB, details on data fields from which we retrieve and derive each metric can be found in Supplementary Tables S2 and S3. Each metric is assessed on a scale of 0–100, with the LE8 score calculated as the mean of these eight individual scores without weighted adjustments. The LE8 score was further categorized into three levels: scores ≥80 (high), scores ≥50 and < 80 (middle), and scores <50 (low).

### *APOE* genotype

The genotype data were assayed with UK BiLEVE Axiom Array and with UK Biobank Axiom™ platforms. Our secondary analysis focused on white individuals or more than 2% missing genotypes processed using PLINK^29^ (version 1.9, www.cog-genomics.org/plink/1.9/). The *APOE* genotype of participants was determined by the two *APOE* isoform coding single nucleotide polymorphisms (SNPs), rs429358 and rs7412, located on chromosome 19. These two SNPs were in Hardy–Weinberg equilibrium (*p*-values > 0.001 for both SNPs) in this cohort. Individuals were categorized as *APOE4*-carriers if they possessed the *APOE* ε3/ε4 or *APOE* ε4/ε4 combinations, while those with the *APOE* ε2/ε2, *APOE* ε2/ε3, *APOE* ε3/ε3 were classified as non-*APOE4*-carriers. *APOE* ε2/ε4 is usually removed because it has both potential risk and protective alleles. We elected for an ε4 dominant (i.e., present vs. absent) rather than a dose model (i.e., 0/1/2), because there were relatively few ε4 homozygotes in our data. The homozygote *APOE* ε2/ε2 has too small sample size (N = 118), the heterozygote *APOE* ε3/ε4 vs. homozygote *APOE* ε4/ε4 showed no significant difference in the main outcome (*p* > 0.05). Thus, when analysing the main effect of *APOE* on BAG, we grouped *APOE* status into *APOE2* carriers (*APOE* ε2/ε2, *APOE* ε2/ε3), *APOE3* homozygotes (*APOE* ε3/ε3), and *APOE4* carriers (*APOE* ε3/ε4 or *APOE* ε4/ε4) for the comparison.

### Main covariates

We included potential confounding factors from baseline in UKB: continuous age, sex (females and males), education (College or University degree and others), Townsend Deprivation Index (an area-based score, continuous), household income (less than £18,000, £18,000–£30,999, £31,000–£51,999, £52,000–£99,999, more than £100,000), moderate alcohol consumption (women: ≤1 unit/day, men: ≤2 unit/day, yes/no), sedentary behaviour (hours of watching television as the proxy), self-related social connection (0-active, 1-moderately active, 2/3-isolated), and baseline health conditions (yes or no: hypertension, cardiovascular diseases, type 2 diabetes, cancer, brain diseases). The missing data rate for education (6.27%), Townsend Deprivation Index (0.085%), household income (6.85%), moderate alcohol consumption (10.55%), television time (6.77%), social connection (0.36%) can be found in Table 1. To address missing covariates, we first assessed whether the missingness is Missing Completely At Random (MCAR) or Missing At Random (MAR). As shown in Supplementary Table S4, the data were partially MCAR; however, for robust statistical inference, we still applied multiple imputations using the chained equations (MICE) method.^30^ The imputation model included WM BAG, LE8 score, and *APOE4* to preserve key relationships in the analysis. The results from multiple imputed datasets were then combined using Rubin’s rules^31^ to ensure valid statistical inference.

**Table 1:** Baseline characteristics of participants in the UK Biobank by overall and categorized life’s essential 8 (LE8) (low, middle, and high) in analysing the main effect of LE8 on the white matter (WM) brain age gap (BAG).

	Level	Overall	Categorized LE8^a^			*p*-value^b^
			Low	Middle	High	
n		18,817	345	14,074	4398	
Age group (%)	40–49	4622 (24.56)	64 (18.55)	2922 (20.76)	1636 (37.20)	<0.001
	50–59	7636 (40.58)	175 (50.72)	5754 (40.88)	1707 (38.81)	
	60–69	6559 (34.86)	106 (30.72)	5398 (38.35)	1055 (23.99)	
Sex (%)	Female	9261 (49.22)	121 (35.07)	6185 (43.95)	2955 (67.19)	<0.001
	Male	9556 (50.78)	224 (64.93)	7889 (56.05)	1443 (32.81)	
Education (%)	College	8731 (46.40)	112 (32.46)	6265 (44.51)	2354 (53.52)	<0.001
	Non-college	8907 (47.33)	193 (55.94)	6812 (48.40)	1902 (43.25)	
	NA	1179 (6.27)	40 (11.59)	997 (7.08)	142 (3.23)	
Household income (%)	Less than £18 k	2049 (10.89)	54 (15.65)	1606 (11.41)	389 (8.84)	<0.001
	£18 k–£31 k	3867 (20.55)	75 (21.74)	3000 (21.32)	792 (18.01)	
	£31 k–£52 k	5252 (27.91)	104 (30.14)	3907 (27.76)	1241 (28.22)	
	£52 k–£100 k	4999 (26.57)	73 (21.16)	3660 (26.01)	1266 (28.79)	
	More than £100 k	1361 (7.23)	16 (4.64)	925 (6.57)	420 (9.55)	
	NA	1289 (6.85)	23 (6.67)	976 (6.93)	290 (6.59)	
Social activity (%)	Active	10,683 (56.77)	152 (44.06)	7863 (55.87)	2668 (60.66)	<0.001
	Moderate active	6707 (35.64)	146 (42.32)	5106 (36.28)	1455 (33.08)	
	Isolated	1359 (7.22)	46 (13.33)	1057 (7.51)	256 (5.82)	
	NA	68 (0.36)	1 (0.29)	48 (0.34)	19 (0.43)	
Moderate Drink (%)	Yes	6020 (31.99)	93 (26.96)	4344 (30.87)	1583 (35.99)	<0.001
	No	10,812 (57.46)	215 (62.32)	8313 (59.07)	2284 (51.93)	
	NA	1985 (10.55)	37 (10.72)	1417 (10.07)	531 (12.07)	
Hypertension (%)	Yes	4645 (24.69)	166 (48.12)	4030 (28.63)	449 (10.21)	<0.001
	No	14,172 (75.31)	179 (51.88)	10,044 (71.37)	3949 (89.79)	
Cardiovascular disease (%)	Yes	1814 (9.64)	50 (14.49)	1487 (10.57)	277 (6.30)	<0.001
	No	17,003 (90.36)	295 (85.51)	12,587 (89.43)	4121 (93.70)	
Diabetes (%)	Yes	907 (4.82)	85 (24.64)	765 (5.44)	57 (1.30)	<0.001
	No	17,910 (95.18)	260 (75.36)	13,309 (94.56)	4341 (98.70)	
Cancer (%)	Yes	2637 (14.01)	49 (14.20)	2052 (14.58)	536 (12.19)	<0.001
	No	16,180 (85.99)	296 (85.80)	12,022 (85.42)	3862 (87.81)	
Brain diseases (%)	Yes	3506 (18.63)	131 (37.97)	2673 (18.99)	702 (15.96)	<0.001
	No	15,311 (81.37)	214 (62.03)	11,401 (81.01)	3696 (84.04)	
Age (mean (SD))		55.45 (7.46)	55.49 (6.55)	56.22 (7.26)	52.98 (7.61)	<0.001
Townsend deprivation Index (mean (SD))		−1.88 (2.70)	−1.05 (3.02)	−1.85 (2.71)	−2.04 (2.64)	<0.001
Sedentary behaviour (TV) (mean (SD))		2.57 (1.41)	3.41 (2.36)	2.65 (1.40)	2.20 (1.23)	<0.001
Diet score (mean (SD))		55.45 (22.89)	36.42 (20.33)	52.41 (22.33)	66.69 (20.73)	<0.001
Smoking score (mean (SD))		69.33 (31.98)	35.19 (30.93)	64.93 (32.15)	86.10 (23.63)	<0.001
Physical activity score (mean (SD))		97.73 (13.18)	69.88 (44.20)	97.81 (12.62)	99.64 (4.11)	<0.001
Sleep score (mean (SD))		90.80 (16.80)	71.25 (27.00)	89.73 (17.43)	95.73 (10.98)	<0.001
BMI score (mean (SD))		72.93 (26.59)	33.61 (24.74)	68.11 (26.35)	91.43 (14.77)	<0.001
nonHDL score (mean (SD))		48.43 (28.61)	24.75 (22.68)	42.56 (25.95)	69.09 (26.91)	<0.001
hba1c score (mean (SD))		93.99 (15.70)	70.00 (27.73)	93.08 (16.50)	98.79 (7.23)	<0.001
BP score (mean (SD))		46.55 (32.42)	19.16 (20.52)	38.49 (28.91)	74.50 (27.24)	<0.001
LE8 score (mean (SD))		71.90 (10.34)	53.18 (13.16)	76.22 (10.98)	87.04 (7.91)	<0.001

a The categorized LE8 score was categorized as high (80–100), middle (50–79), and low (0–49).

b *p*-values were calculated with an ANOVA and Chi-square test for continuous and categorical variables, respectively.

### Statistical analysis

We computed descriptive characteristics of the LE8-BAG analytical sample categorized by LE8 scores (low, middle and high) and the Gene–Environment interaction analytical sample categorized by *APOE4* status (carriers and non-carriers). The distribution of the variables among the groups was compared using the χ^2^ test for categorical variables and ANOVA tests for numerical variables.

We applied the random forest (RF) regression method to predict WM brain age and estimate WM BAG from the FA data. We have tested other machine learning approaches (e.g., support vector regression, elastic net, and gradient boosting, among others) and RF was selected as the final model for its most superior predictive performance.^14^^,^^32^ Additionally, WM BAG estimation results from different machine learning algorithms were highly similar, indicating that the estimation process is largely independent of the specific modelling approach.^32^ We followed a similar data splitting scheme as our previous studies to split the data into non-overlapping training and testing set.^13^^,^^14^ First, we trained a RF regression model with regional FA measures as predictors and chronological age as outcome to estimate brain age in the training set of non-disease participants (those who were not previous or current smokers, and did not have hypertension, cardiovascular disease, diabetes, or brain diseases, see Supplementary Table S5 for the detailed ICD codes). The RF regression model includes a two-step feature selection and ranks the features by importance scores to identify the most important FA tracts contributing to BAG prediction.^33^ The parameters of the RF regression were tuned based on predictive performance criteria: the coefficients of determination (R) and mean absolute error (MAE), using 5-fold cross-validation. Then, the final RF model was locked and applied to the testing samples including both disease and non-disease participants to predict their brain age. The WM BAG was calculated by subtracting individuals’ chronological age from their predicted brain age. Age-dependent bias has been found in brain age estimates across many clinical studies.^34^ To address this, we applied a simple regression model to remove the brain age prediction bias from WM BAG.

We investigated the main effects of total LE8 score (0–100), individual LE8 components, categorized LE8 score (low, middle, high), as well as the main effect of *APOE* status, on WM BAG using general linear regression models. Model 1 adjusted for age and sex. Model 2 further adjusted for education, Townsend deprivation index, moderate alcohol consumption, sedentary behaviour, and social connection. Model 3 further adjusted for the prevalent diseases. The inverse probability weighting (IPW) method was employed as an alternative method to mitigate the risk of confounding and provide potential causal evidence for the association between categorized LE8 score and WM BAG. To further identify which region of the brain is most sensitive to the effects, we also performed regression analyses of each FA tract on LE8 or *APOE4* using Model 3. We then investigated the potential interaction between lifestyle and *APOE4* to see how *APOE4* gene might alter the effect of LE8 on WM BAG. We also evaluated the effect of LE8 on WM BAG stratified by *APOE4* status. All these analyses were further stratified by baseline age (40–49, 50–59, 60–69) and sex (female or male).

As white matter brain ageing is closely related to risk for neurodegenerative diseases, we also compared the risk ratios of all-cause dementia (definitions in Supplementary Table S6) in different groups classified by genetic and lifestyle factor. We calculated crude and age-adjusted risk ratios (RR) for all-cause dementia among women and men in four distinct groups, stratified by *APOE4* carrier status (carriers and non-carriers) and Life’s Essential 8 (LE8) scores (low: <70; high: ≥ 70). The group of non-*APOE4* carriers with low LE8 scores was regarded as the reference category.

All statistical analyses were conducted using R (version 4.0.5). R packages, including “*MICE*” (version 3.14.0), “*Hmisc*” (version 5.1.0) were used to perform multiple imputation by chained equations and inverse probability weighting analyses.

### Role of funders

The funders were not involved in the study design, data analysis and interpretation of results or writing of this manuscript.

## Results

The overall sample used to examine the association between LE8 and the BAG included 18,817 white participants with a mean (SD) age of 55.45 (7.46) years of whom 49.22% were women. The mean (SD) LE8-total score (ranging from 0 to 100) was 71.90 (10.34). Participants in the middle and high LE8 group (≥80) were younger, more likely to be female, to have a college degree, greater household income, lower Townsend deprivation index, greater active social activity, and more likely to report “no or moderate” alcohol consumption compared to the low LE8 group (<50) (Table 1). In the Gene–Environment interaction sub-sample (N = 18,259) exploring the effect modification of the *APOE4* allele, 25.82% were *APOE4* carriers (Supplementary Table S7). *APOE4* carriers were younger, more often women, and with lower lipid levels but higher HbA1c and blood pressure levels (Supplementary Table S7).

### Estimation of the outcome WM BAG

The optimal random forest regression model selected 16 FA measures for BAG estimation (Supplementary Table S1). Our selected tracts, including the fornix (FX, FXST-L/R), corpus callosum fibers (GCC, SCC, BCC), and projection pathways (ACR, PCR, PTR, PLIC, ALIC), suggest that limbic system integrity, interhemispheric connectivity, and deep white matter pathways play a crucial role in BAG prediction, highlighting their importance in brain ageing and neurodegeneration. After correcting for age bias on the white matter BAG, the adjusted predicted BAG achieved R = 0.89, MAE = 2.74 years in both the disease test dataset and the non-disease test dataset (Supplementary Fig. S1). The disease group was, on average, 0.28 years (95%CI = 0.19 to 0.36; *p* = 4.91E-10) older in WM brain age than the non-disease group.

### Main effect of LE8 on WM BAG

**Fig. 3:**
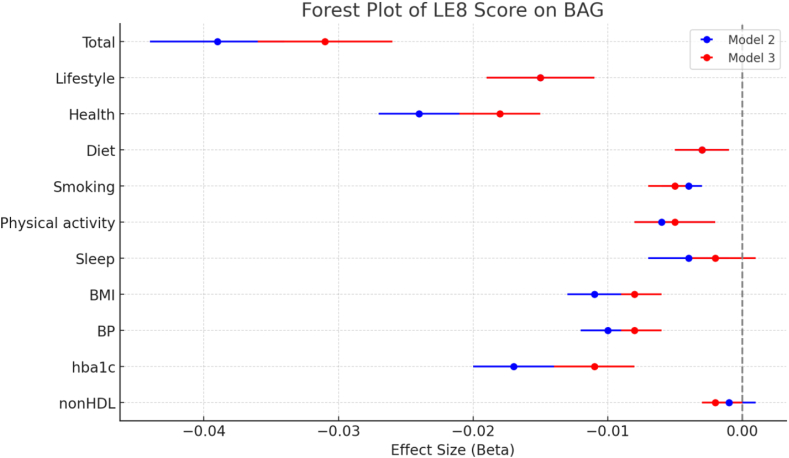
Forest plot of the effect sizes with 95% confidence intervals for joint and individual life’s essential 8 (LE8) scores on white matter brain age gap (BAG) in models 2 and 3. Model 2: model 1+ education, Townsend deprivation index, moderate drink, sedentary behaviour, social connection. Model 3: model 2+ medical conditions (hypertension, cardiovascular disease, cancer, brain diseases). BMI: body mass index. BP: blood pressure.

The regression analyses revealed significant associations between LE8 and WM BAG for both continuous and categorized LE8 (Table 2). Higher LE8 score was associated with a lower WM BAG (β_model1_ = −0.042, β_model2_ = −0.039, β_model3_ = −0.031, all *p* < 0.001), indicating delayed brain ageing (153-, 142- and 113-days younger brain age per 10-point increase for model 1, 2 and 3, respectively). The middle LE8 group (β = −0.88 (−321 days), 95% CI: −1.23 to −0.53 (−449 to −193 days), *p* < 0.001) and the high LE8 group (β = −1.34 (−489 days), 95% CI: −1.71 to −0.99 (−624 to −361 days), *p* < 0.001) showed a significantly lower WM BAG compared to the low LE8 group (as reference) in model 3. This pattern was held across all models, adjusting for demographics, socioeconomic status, and medical conditions. IPW analysis validated the effect of LE8 groups on WM BAG from our main analysis and provided potentially causal evidence (Supplementary Table S8). Consistent beneficial associations in all models were also observed for individual LE8 factors including a favourable diet, no smoking, higher level of physical activity, favourable BMI, and normal blood pressure and HbA1c levels (Supplementary Table S9 and Fig. 3).

**Table 2:** Main effects of 1) joint life’s essential 8 (LE8) and 2) *APOE* status on white matter Brain Age Gap by using the general linear regression model.

	Model 1		Model 2		Model 3	
1) LE8						
LE8 Score (Continuous)	beta (95% CI)	*p*	beta (95% CI)	*p*	beta (95% CI)	*p*
Total (0–100)	−0.042 (−0.046 to −0.037)	<0.001	−0.039 (−0.044 to −0.034)	<0.001	−0.031 (−0.036 to −0.026)	<0.001
Categorized LE8∗	beta (95% CI)	*p*	beta (95% CI)	*p*	beta (95% CI)	*p*
Low (<50)	Ref		Ref		Ref	
Middle (≥50 and < 80)	−1.294 (−1.640 to −0.948)	<0.001	−1.213 (−1.561 to −0.866)	<0.001	−0.882 (−1.230 to −0.534)	<0.001
High (≥80)	−1.914 (−2.270 to −1.558)	<0.001	−1.775 (−2.134 to −1.416)	<0.001	−1.343 (−1.705 to −0.982)	<0.001
2) *APOE*						
*APOE* status-2 groups	beta (95% CI)	*p*	beta (95% CI)	*p*	beta (95% CI)	*p*
Non *APOE4* carriers (2/2, 2/3, 3/3)	Ref		Ref		Ref	
*APOE4* carriers (3/4, 4/4)	0.100 (0.014–0.186)	0.022	0.101 (0.016–0.187)	0.021	0.110 (0.025–0.195)	0.011
*APOE* status-3 groups	beta (95% CI)	*p*	beta (95% CI)	*p*	beta (95% CI)	*p*
*APOE* 3/3	Ref		Ref		Ref	
*APOE4* carriers (3/4, 4/4)	0.092 (0.003–0.180)	0.042	0.094 (0.005–0.182)	0.038	0.104 (0.016–0.191)	0.020
*APOE2* carriers (2/2,2/3)	−0.046 (−0.159 to 0.066)	0.419	−0.042 (−0.154 to 0.071)	0.467	−0.034 (−0.145 to 0.078)	0.552

95% CI: 95% confidence interval.

Model 1: age, sex.

Model 2: model 1+ education, Townsend deprivation index, moderate drink, sedentary behaviour, social connection.

Model 3: model 2+ medical conditions (hypertension, cardiovascular disease, cancer, brain diseases).

The steepest decline in WM BAG with increasing LE8 score was observed in the 50–59 age group (Supplementary Fig. S2A, *p* for interaction (40–49 vs. 50–59) <0.001). There was no sex difference in the association between LE8-total score and WM BAG in each age group (all *p*_interaction_ > 0.05) (Supplementary Fig. S2B).

Linear regression models evaluating the association between each DTI metric and LE8 found significant associations in a majority of tracts for each metric, where FA consistently exhibited the strongest effect size (Supplementary Table S10), reinforcing its selection as the primary metric for our analysis. Certain white matter tracts are more sensitive to the effects of LE8 (e.g. FXST, GCC and ACR, etc.; see z-score plot in Supplementary Fig. S3 ordered by the effect sizes) than the others.

### Main and modification effect of *APOE4* genotype on WM BAG

*APOE4* carriers exhibited a significantly greater WM BAG compared to non-*APOE4* carriers across all three models (β_model1_ = 0.10, β_model2_ = 0.10, β_model3_ = 0.11, *p* = 0.02, 0.02 and 0.01), indicating accelerated brain ageing (37-, 37- and 40-days older brain in *APOE4* carriers for model 1, 2 and 3, respectively). In contrast, *APOE3/3* and *APOE2* carriers (ε2/ε2, ε2/ε3) did not show any significant difference in WM BAG (Table 2). Therefore, for subsequent analyses, we only focused on the comparison between *APOE4* carriers and non-carriers. Per-tract analysis identified CHG, ICP and PTR tracts more prone to genetic influence of *APOE4* (see z-score plot in Supplementary Fig. S3).

**Fig. 4:**
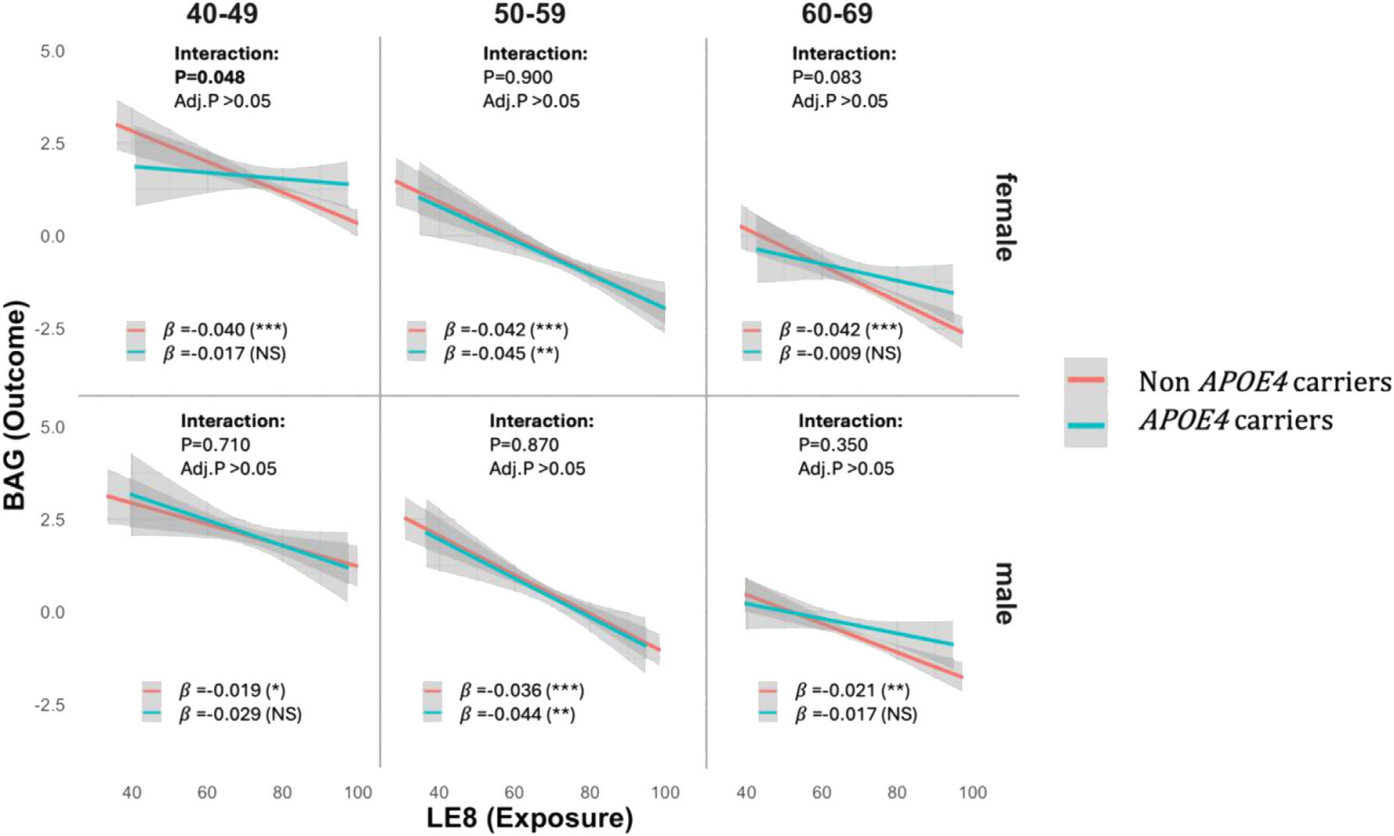
Interaction effects of *APOE4* and Life’s Essential 8 (LE8) scores on white matter (WM) brain age gap (BAG) across different age and sex groups. The graphs present both unadjusted *p*-values and FDR-adjusted *p*-values (Benjamini-Hochberg correction) for testing the interaction between LE8 and *APOE4*. Additionally, the regression effects of LE8 on WM BAG are shown separately for *APOE4* carriers and non-carriers at the bottom of each section (β coefficients are derived from models adjusted for age, education, Townsend Deprivation Index, moderate alcohol consumption, sedentary behaviour, social connections, and medical conditions). Statistical significance is denoted as follows: NS (non-significant), ∗ (Adjusted *p* < 0.05), ∗∗ (Adjusted *p* < 0.01), and ∗∗∗ (Adjusted *p* < 0.001).

The modification effects of the *APOE4* genotype revealed a complex pattern. No overall interaction between LE8 and *APOE4* status was observed; however, a few single LE8 component x *APOE4* interactions showed marginal significance among women or men (*p* = 0.029 for BMI x *APOE4* in women and *p* = 0.036 for Diet x *APOE4* in men; Supplementary Table S11). When stratified by sex and age groups, we observed a significant LE8 x *APOE4* interaction in women aged 40–49 (*p* = 0.048) but the significance disappeared after multiple comparison adjustment (Fig. 4). Stratified analyses by *APOE4* status further revealed that non-*APOE4* carriers consistently benefited from higher LE8 scores with reduced WM BAG across all age groups (124 days younger per 10-point increase, 95% CI: 102–146 days; *p* < 0.001; Supplementary Tables S12 and S13). In contrast, *APOE4* carriers exhibited variable and generally weaker responses (84 days younger per 10-point increase, 95% CI: 47–120 days; *p* < 0.001; Supplementary Tables S12 and S13). While the interaction effect was not statistically significant after adjustment, the observed stratification patterns suggest that the influence of health behaviours on brain ageing may differ by *APOE4* status, sex and age groups. These findings indicate potential gene-lifestyle interactions that warrant further investigation in larger and more diverse cohorts.

### Risk ratios of dementia among *APOE4* and LE8 combined groups

Overall, *APOE4* carriers had a higher risk of all-cause dementia in women and men compared to non-*APOE4* carriers. *APOE4* carriers with high LE8 (≥70) in women (crude RR: 0.62, *p* < 0.001) and men (crude RR: 0.82, *p* = 0.003) showed a significant reduction in all-cause dementia compared with those with low LE8 (<70), though this association lost significance after age adjustment (Supplementary Fig. S4 and Table S14).

## Discussion

Our study, conducted using rich genetic, imaging, and other health-related data from the UKB, advances the understanding of how LE8 is associated with white matter brain ageing and how this effect is modified by *APOE4* status in a large prospective cohort in the UK. Our findings highlight that higher LE8 scores were associated with delayed white matter brain ageing. This was further influenced by the *APOE4* allele’s modification effects, where non-*APOE4* carriers showed a consistent delayed brain ageing across all levels of LE8, and *APOE4* carriers displayed diminished effects. Notably, our findings highlighted some sex and age difference in the association between LE8 and brain ageing particularly among *APOE4* carriers, which is worth further investigation in larger and more targeted studies in the future.

Our findings linking LE8 to delayed white matter brain ageing have not been previously described and were built on prior studies that found associations of healthy behaviours/lifestyles (LS7, LE8, and related) with better cognition, lower dementia risk, and better micro-/macro-brain structures.^2^^,^^35^, ^36^, ^37^ Collectively, our findings underline the importance of adherence to a healthy lifestyle and behaviour in maintaining brain integrity across various demographics and ages, underscoring the long-term value of LE8 and WM BAG as reliable markers in ageing studies.^38^^,^^39^ These markers effectively monitor the sustained impacts of lifestyle on brain health, highlighting the importance of promoting healthier lifestyle in reducing neurodegenerative risk. While the *APOE4* x LE8 interaction is weak and a higher LE8 score is associated with a lower WM BAG regardless of *APOE4* status, we observed an attenuated effect size of LE8 in *APOE4* carriers. The relatively weaker and more variable LE8 effect implies a potential limit to the neuroprotective effects of the lifestyle factors in individuals carrying the *APOE4* allele, suggesting a comprehensive, multi-factorial lifestyle approach may be more effective than emphasizing a single intervention for *APOE4* carriers. We have previously highlighted the moderating effect of *APOE4* on the relationship between plasma metabolites and white matter microstructural integrity,^40^ where low-density lipoprotein detrimentally affects white matter integrity in critical neural tracts among *APOE4* carriers, which may be plausible biological mechanism underlying the interaction between *APOE4* and LE8.

Additionally, though relatively weak, our findings suggest that sex and age differences may modulate the relationship between *APOE4*, inflammation, and brain ageing, potentially influencing how lifestyle factors interact with genetic risk. Prior research indicates that *APOE4* carriers exhibit distinct inflammatory and metabolic responses based on sex, with women generally showing greater susceptibility to tau pathology, along with neuroinflammation and metabolic dysfunction.^41^, ^42^, ^43^ This may explain why *APOE4* has been linked to a higher risk of Alzheimer’s disease in women, particularly postmenopausal, when oestrogen—known for its neuroprotective and anti-inflammatory effects—declines.^44^ Sex differences in *APOE4*’s interaction with lipid metabolism have been observed in animal studies, with male *APOE4* mice showing more pronounced disruptions in lipid processing and increased neuroinflammation on a high-fat diet compared to females.^45^^,^^46^ These mechanistic differences may underlie the weak but suggestive interactions observed in our study, where BMI × *APOE4* in women and Diet × *APOE4* in men showed marginal significance before multiple comparison adjustments. Furthermore, we observed more noticeable LE8 x *APOE4* interaction effect in the 40–49 and 60–69 age groups (especially among women) than in the 50–59 group, suggesting age-dependent shifts in *APOE4*-related inflammatory and metabolic pathways. Collectively, these findings highlight the importance of considering sex and age when evaluating lifestyle-genetic interactions in brain ageing and suggest that future studies should investigate whether personalized lifestyle interventions could mitigate *APOE4*-related neurodegeneration across different demographic groups.

Our study has several strengths. Firstly, it leverages large-scale imaging, genetic and health-related data from questionnaires, physical examinations, and biological samples in the UK Biobank. Second, using machine learning to compute biological brain age introduces an innovative and potentially more accurate method of assessing brain ageing, allowing us to detect subtle changes in brain ageing that traditional methods might overlook. Additionally, we offer comprehensive LE8 metrics, including a wide range of modifiable behaviours and health factors, as a holistic approach. Moreover, the inclusion of the *APOE4* modification effect together with age- and sex-stratified analysis addresses an important aspect of personalized and precision medicine. Lastly, our approach is strengthened by using robust and rigorous statistical tools, including the IPW method.

This study has several limitations. First, potential selection bias may arise due to the relatively healthy volunteers in the UK Biobank. Second, reliance on self-reported questionnaires for certain lifestyle factors could introduce recall or misclassification bias. Additionally, unmeasured factors such as environmental exposures and psychosocial stressors may influence outcomes. Third, the cross-sectional design limits our ability to infer causal relationships, as LE8 and brain ageing may influence each other over time. Further research is needed to determine whether lifestyle modifications actively influence brain ageing trajectories. Future studies should leverage longitudinal cohorts, such as the Adolescent Brain Cognitive Development study and the Baltimore Longitudinal Study of ageing investigate these dynamic relationship, as well as randomized controlled trials (RCTs) and intervention-based studies to help assess the potential benefits of targeted lifestyle interventions on brain health. In addition, our analysis is restricted to white participants in the UK Biobank limiting its generalizability, expanding research to more diverse populations such as those in All of Us is necessary in future studies. Lastly, our current study has looked at the LE8 effect and its interaction with *APOE4,* a genetic risk factor specific to dementia, on white matter brain ageing. Future research should also explore the impact of LE8 on other age-related brain disorders, such as Parkinson’s disease or depression, while considering its interaction with other specific genetic risk factors, potentially broadening the application value of LE8 in neurodegenerative and mental health research.

### Conclusion

Our research provides evidence that Life’s Essential 8, is vital in slowing the progression of white matter brain ageing among participants of European ancestry in the UK Biobank. The public health implications of our findings are profound, suggesting overall benefit from a healthier lifestyles in individuals’ brain ageing across genetic, sex, and age groups, highlighting the broad applicability of behavioural lifestyle interventions in promoting brain health.

## Contributors

LF took the lead in performing the analysis and wrote the manuscript. ZY and YP processed the genetic and imaging data. LF, TM and SC conceptualized the idea. TM and SC supervised the project, took the lead in editing the manuscript, and acquired the funding. RGM, BDM, PK, PMT, JC, ML, TN, ES, YL, TC, HK, HL, SL, EH, CC and DL contributed to manuscript writing and polishing. All authors provided critical feedback and helped to shape the research, analysis, and manuscript. All authors read and approved the final version of the manuscript, and ensure it is the case. Access and verification of underlying data: LF and ZY.

## Data sharing statement

The raw genetic and phenotypic data used for this study can be found in the UK Biobank (http://www.ukbiobank.ac.uk/).

## Declaration of interests

None.
